# Supplementary material for: HIVprotI: an integrated web based platform for prediction and design of HIV proteins inhibitors
Source: J Cheminform. 2018 Mar 9;10:12. doi: 10.1186/s13321-018-0266-y (PMC5845081; doi:10.1186/s13321-018-0266-y)
Supplement: Supplementary file 2 — Additional file 2. Source code of HIVProtI web server. [file 13321_2018_266_MOESM2_ESM.zip › HIVprotI_Source-code/hivprotI/weka-3-6-6/doc/weka/experiment/Stats.html]

Stats


---


|  |  |  |
| --- | --- | --- |
| **Overview** \| **Package** \| **Class** \| **Tree** \| **Deprecated** \| **Index** \| **Help** \| **Weka's home** | |  |
| **PREV CLASS**   **NEXT CLASS** | **FRAMES**    **NO FRAMES**     **All Classes** |  |
| SUMMARY: NESTED \| FIELD \| CONSTR \| METHOD | DETAIL: FIELD \| CONSTR \| METHOD |  |


---


## weka.experiment Class Stats

```
java.lang.Object
  weka.experiment.Stats
```

**All Implemented Interfaces:**: java.io.Serializable, RevisionHandler

---

``` public class Stats extends java.lang.Object implements java.io.Serializable, RevisionHandler ```

A class to store simple statistics

**Version:**
:   $Revision: 1.12 $

**Author:**
:   Len Trigg (trigg@cs.waikato.ac.nz)

**See Also:**: Serialized Form

---

| **Field Summary** | |
| --- | --- |
| `double` | `count`             The number of values seen |
| `double` | `max`             The maximum value seen, or Double.NaN if no values seen |
| `double` | `mean`             The mean of values at the last calculateDerived() call |
| `double` | `min`             The minimum value seen, or Double.NaN if no values seen |
| `double` | `stdDev`             The std deviation of values at the last calculateDerived() call |
| `double` | `sum`             The sum of values seen |
| `double` | `sumSq`             The sum of values squared seen |


**Constructor Summary**

`Stats()`


| **Method Summary** | |
| --- | --- |
| `void` | `add(double value)`             Adds a value to the observed values |
| `void` | `add(double value, double n)`             Adds a value that has been seen n times to the observed values |
| `void` | `calculateDerived()`             Tells the object to calculate any statistics that don't have their values automatically updated during add. |
| `java.lang.String` | `getRevision()`             Returns the revision string. |
| `static void` | `main(java.lang.String[] args)`             Tests the paired stats object from the command line. |
| `void` | `subtract(double value)`             Removes a value to the observed values (no checking is done that the value being removed was actually added). |
| `void` | `subtract(double value, double n)`             Subtracts a value that has been seen n times from the observed values |
| `java.lang.String` | `toString()`             Returns a string summarising the stats so far. |

**Methods inherited from class java.lang.Object**

`equals, getClass, hashCode, notify, notifyAll, wait, wait, wait`

**Field Detail**

### count

```
public double count
```

:   The number of values seen

---


### sum

```
public double sum
```

:   The sum of values seen

---


### sumSq

```
public double sumSq
```

:   The sum of values squared seen

---


### stdDev

```
public double stdDev
```

:   The std deviation of values at the last calculateDerived() call

---


### mean

```
public double mean
```

:   The mean of values at the last calculateDerived() call

---


### min

```
public double min
```

:   The minimum value seen, or Double.NaN if no values seen

---


### max

```
public double max
```

:   The maximum value seen, or Double.NaN if no values seen


**Constructor Detail**

### Stats

```
public Stats()
```


**Method Detail**

### add

```
public void add(double value)
```

:   Adds a value to the observed values

    :   **Parameters:**: `value` - the observed value

---


### add

```
public void add(double value,
                double n)
```

:   Adds a value that has been seen n times to the observed values

    :   **Parameters:**: `value` - the observed value: `n` - the number of times to add value

---


### subtract

```
public void subtract(double value)
```

:   Removes a value to the observed values (no checking is done
    that the value being removed was actually added).

    :   **Parameters:**: `value` - the observed value

---


### subtract

```
public void subtract(double value,
                     double n)
```

:   Subtracts a value that has been seen n times from the observed values

    :   **Parameters:**: `value` - the observed value: `n` - the number of times to subtract value

---


### calculateDerived

```
public void calculateDerived()
```

:   Tells the object to calculate any statistics that don't have their
    values automatically updated during add. Currently updates the mean
    and standard deviation.

---


### toString

```
public java.lang.String toString()
```

:   Returns a string summarising the stats so far.

    :   **Overrides:**: `toString` in class `java.lang.Object`
    :   **Returns:**: the summary string

---


### getRevision

```
public java.lang.String getRevision()
```

:   Returns the revision string.

    :   **Specified by:**: `getRevision` in interface `RevisionHandler`
    :   **Returns:**: the revision

---


### main

```
public static void main(java.lang.String[] args)
```

:   Tests the paired stats object from the command line.
    reads line from stdin, expecting two values per line.

    :   **Parameters:**: `args` - ignored.


---


|  |  |  |
| --- | --- | --- |
| **Overview** \| **Package** \| **Class** \| **Tree** \| **Deprecated** \| **Index** \| **Help** \| **Weka's home** | |  |
| **PREV CLASS**   **NEXT CLASS** | **FRAMES**    **NO FRAMES**     **All Classes** |  |
| SUMMARY: NESTED \| FIELD \| CONSTR \| METHOD | DETAIL: FIELD \| CONSTR \| METHOD |  |


---
